# Propolis Modifies Collagen Types I and III Accumulation in the Matrix of Burnt Tissue

**DOI:** 10.1155/2013/423809

**Published:** 2013-05-28

**Authors:** Pawel Olczyk, Grzegorz Wisowski, Katarzyna Komosinska-Vassev, Jerzy Stojko, Katarzyna Klimek, Monika Olczyk, Ewa M. Kozma

**Affiliations:** ^1^Department of Community Pharmacy, Medical University of Silesia in Katowice, 41-200 Sosnowiec, Poland; ^2^Department of Clinical Chemistry and Laboratory Diagnostics, Medical University of Silesia in Katowice, 41-200 Sosnowiec, Poland; ^3^Center of Experimental Medicine, Medical University of Silesia in Katowice, 40-752 Katowice, Poland; ^4^Department of Statistics, Medical University of Silesia in Katowice, 41-200 Sosnowiec, Poland

## Abstract

Wound healing represents an interactive process which requires highly organized activity of various cells, synthesizing cytokines, growth factors, and collagen. Collagen types I and III, serving as structural and regulatory molecules, play pivotal roles during wound healing. The aim of this study was to compare the propolis and silver sulfadiazine therapeutic efficacy throughout the quantitative and qualitative assessment of collagen types I and III accumulation in the matrix of burnt tissues. Burn wounds were inflicted on pigs, chosen for the evaluation of wound repair because of many similarities between pig and human skin. Isolated collagen types I and III were estimated by the surface plasmon resonance method with a subsequent collagenous quantification using electrophoretic and densitometric analyses. Propolis burn treatment led to enhanced collagens and its components expression, especially during the initial stage of the study. Less expressed changes were observed after silver sulfadiazine (AgSD) application. AgSD and, with a smaller intensity, propolis stimulated accumulation of collagenous degradation products. The assessed propolis therapeutic efficacy, throughout quantitatively and qualitatively analyses of collagen types I and III expression and degradation in wounds matrix, may indicate that apitherapeutic agent can generate favorable biochemical environment supporting reepithelization.

## 1. Introduction

Propolis, a naturally occurring resinous substance, represents a popular remedy, well known for its broad spectrum of biological activities including antimicrobial, antioxidant, anaesthetic, anti-inflammatory, and immune-modulatory actions [1–5]. The apitherapeutic agent, which is easily available and well tolerated with rare incidents of allergy and no toxicity, is referred to as an excellent candidate for burn management, enhancing fibroblasts proliferation, activation, and growth capacity [1, 6–8]. Nowadays, silver sulfadiazine (AgSD) used as an agent of choice in the treatment of burn wound, due to broad spectrum of antimicrobial activity, can also be responsible for certain considerable adverse reactions [9]. AgSD may not only contribute to the development of argyria, inner organ dysfunction (liver, spleen, and kidney) due to silver systemic accumulation or determined by sulphadiazine presence, dermatitis, erythema multiforme rashes, and acute hemolytic anemia but also, unfortunately, could be responsible for cytotoxic effect on fibroblasts and keratinocytes [9, 10]. Described cytotoxic influence may efficiently retard wound healing process, fundamental response on tissue injury (comprised of four precisely integrated phases, such as hemostasis, inflammation, proliferation, and remodeling)—requiring highly coordinated activity of various cells [10–14]. Fibroblasts and keratinocytes seem to play pivotal roles during wound healing, since their interactions participate in changing the wound environment from an inflammatory to a synthesis-driven granulation tissue [15–17]. Moreover, while migrating from the wound margin and proliferating keratinocytes, which are involved in reepithelization [6], fibroblasts, differentiated into myofibroblasts and involved in wound contraction and extracellular matrix (ECM) remodeling, are responsible for the production of ECM components including proteoglycans, glycosaminoglycan, elastin, and collagen [10]. Collagen as a structurally and functionally pivotal molecule, which builds a scaffold in the connective tissue, is also involved in hemostasis, inflammatory response, cell growth, differentiation, and migration [11, 18, 19]. Furthermore, collagen participates in cell signaling, angiogenesis, expression of inflammatory cytokines and growth factors, and interactions between matrix metalloproteinases (MMPs) and their tissue inhibitors being the inherent element of reepithelization [11, 18, 19]. Taking into account that collagen types I and III are the main collagen types of healthy skin, being predominantly expressed during repair process [19], the aim of this study was to compare the propolis and silver sulfadiazine therapeutic efficacy (in the treatment of thermal injuries) throughout the quantitative and qualitative assessment of the mentioned collagen types accumulation in the matrix of burnt tissues.

## 2. Material and Methods

### 2.1. Reagents and Materials

The following antibodies were used: polyclonal rabbit anti-human collagen type III antibodies (Rockland, Gilbertsville, PA, USA; no. 600-401-105-0.1), goat anti-rabbit immunoglobulin G conjugated with horseradish peroxidase (Sigma-Aldrich, Germany; no. A5420), and mouse monoclonal antibody raised against full length native soluble acid digested pepsin collagen type I of human origin (Santa Cruz Biotechnology Inc., CA, USA; no. sc-59773). The following reagents were used: sodium metaperiodate and hydrazide LC-biotin obtained from Thermo Scientific, USA; DMSO (dimethylsulfoxide), sodium dodecyl sulfate, Triton X-100, Coomassie brilliant blue R250, pepsin, glycine, Immobilon P membranes, dithiothreitol, Tween 20 (polyoxyethylenesorbitan monolaurate), TMB (3.3′,5,5′-tetramethylbenzidine), standard type I collagen from calf skin, and standard type III collagen from the human placenta purchased from Sigma-Aldrich, Germany; Sephadex G-25 obtained from Pharmacia, Sweden; HEPES (4-(2-hydroxyethyl) piperazine-1-ethanesulfonic acid) supplied by Fluka, Germany; BLOT-QuickBlocker purchased from Millipore, USA. All the remaining reagents were supplied by POCh, Poland.

### 2.2. Therapeutic Agents

Propolis formulation (apitherapeutic ointment) was accepted by the National Institute of Hygiene (certificate number: HZ/06107/00; date: November 4, 2000), 1% (0.01 g/mL) silver sulfadiazine (AgSD) cream, Lek Poland.

### 2.3. Tissue Materials

The study protocol was approved by the Ethics Committee of the Medical University of Silesia, Poland. Four 16-week-old, domestic pigs were chosen for the evaluation of wound repair because of many similarities between pig and human skin. Seventy-two contact burn wounds were inflicted on the right and left flanks of the pig body, according to the methods of Hoekstra et al. [20] and Brans et al. [21]. Pigs were housed according to the Good Laboratory Practice (GLP) Standards of Polish Veterinary Law. Animals were divided into control (*n* = 2) and experimental (*n* = 2) groups. In the control group, wounds were treated with physiologic saline (NaCl) (one animal) or with a propolis vehicle (another animal) twice a day, for 21 days. Wounds treated with NaCl allowed to observe the healing process occurring without management. Wounds treated with the vehicle alone allowed, in turn, to assess its possible impact on the propolis therapeutic effect. In the experimental groups, burns were treated with propolis (one animal) or AgSD (another animal), twice a day, for 21 days. Biopsies, in three replications, were taken from healthy skin at day 0 and from the different wound beds on postburn days 3rd, 5th, 10th, 15th, and 21st. Analgesics given were ketamine hydrochloride and thiopental sodium. Following thermal damage, tissues were rinsed with an antiseptic solution and treated with apitherapeutic agent, AgSD, apitherapeutic agent vehicle, and physiologic saline. In the case of burns treated with the apitherapeutic agent, AgSD, and apitherapeutic agent vehicle, the wound beds were covered with 55–75 mm layer of used experimental agents. Then, the injuries were protected with a woven cotton material. The thermal injuries left by the biopsy were protected with the collagen dressing.

### 2.4. Extraction of Tissue Collagen Types I and III

Tissue samples, each weighing 10 mg, after homogenization with acetone, were suspended in 0.5 M acetic acid and subjected to pepsin action for 24 h at 4°C with shaking. Tissue lysates were then centrifuged (30 000 r/min, 4°C for 30 min) and obtained supernatants were collected while tissue pellets were repeatedly extracted as described above [22]. Combined supernatants were lyophilized.

### 2.5. Biotinylation of Antibodies against Collagen Types I and III

Antibodies against collagen types I and III were dissolved in 0.1 M acetate buffer (pH = 5.5). The obtained solutions were cooled and protected from light and then mixed with an equal volume of 0.02 M sodium metaperiodate in 0.1 M sodium acetate buffer (pH = 5.5). Subsequently, the samples were incubated for 0.5 h at 4°C and then subjected to gel filtration on Sephadex G-25 equilibrated in PBS buffer, pH = 7.2. 1 mL fraction eluting at column void volume and containing anticollagen antibodies were collected and mixed with 111 *μ*L of 0.05 M solution of hydrazide LC-biotin in DMSO. Biotinylation of antibodies was conducted for 2 h, at room temperature. Next, free biotin was removed by dialysis against distilled water and modified anticollagen antibodies were lyophilized. The efficiency of antibody biotinylation was controlled with special EZ biotin quantitation kit (Thermo Scientific).

### 2.6. Quantification of Collagen Types I and III in the Hydrolyzates of Burn Wounds

The assessment of collagen content in the tissue material derived from healing postburn wounds was made by surface plasmon resonance (SPR) measurement in SPRINGLE Instrument (Autolab, the Netherlands) [23, 24]. For this purpose, the biotinylated anticollagen antibodies were immobilized onto streptavidin-coated sensor chip (SAP from XanTec, Germany) and exposed at 21°C to components released from tissue by pepsin. The binding was conducted in 0.01 M HEPES buffer, pH 7.4, containing 0.0034 M EDTA, 0.15 M NaCl, and 0.05% (v/v) Triton X-100. The formation of complexes between the antibody and collagen was detected as changes in the SPR signals which were proportional to the amount of bound collagen molecules. After binding, the disc surface was regenerated through the dissociation of immune complexes with 0.01 M glycine solution, pH 2.0. The calibration curves were done using various concentration of standard collagen types I and III.

### 2.7. The Assessment of Type I Collagen Components in Burn Wounds

To assess the profile of type I collagen species released by pepsin from healing wounds, the components of tissue hydrolyzates were subjected to sodium dodecyl sulfate-gradient polyacrylamide gel electrophoresis (SDS-PAGE) at nonreducing conditions, according to Laemmli method [25]. Gels were stained with Coomassie brilliant blue R-250. To evaluate the interference of type III collagen in obtained electrophoretic patterns, some gels were submitted to alternative procedure containing electrotransfer of resolved collagen compounds to Immobilon-P membranes followed by immunoblotting (probing) with anti-type III collagen serum as described below.

### 2.8. The Assessment of Type III Collagen Components in Burn Wounds

The profile of type III collagen compounds generated from healing wounds by pepsin was estimated by SDS-gradient PAGE followed by western blotting with appropriate serum. Briefly, components of tissue hydrolyzates were resolved on 4–15% SDS-PAGE in the presence of 0.04 M dithiothreitol as the agent which reduces disulfide bonds and then electrotransferred to Immobilon-P membranes. Subsequently, the membranes were blocked for 1 h in 5% BLOT-QuickBlocker solution prepared in 0.05 M Tris-HCl buffer, pH 7.4 containing 0.15 M NaCl and 0.1% (v/v) Tween 20 (TBS buffer). Then, the membranes were probed overnight at 4°C with polyclonal rabbit anti-type III collagen antibodies diluted 1 : 5 000 with TBS. Horseradish peroxidase, conjugated goat anti-rabbit IgG diluted 1 : 50 000 with TBS, was used as a secondary antibody. 

### 2.9. Statistical Analysis

Statistical differences between groups were determined by a multivariate analysis of variance (ANOVA), followed by Tukey's post hoc tests, accepting *P* < 0.05 as significant.

## 3. Results

**Figure 1:**
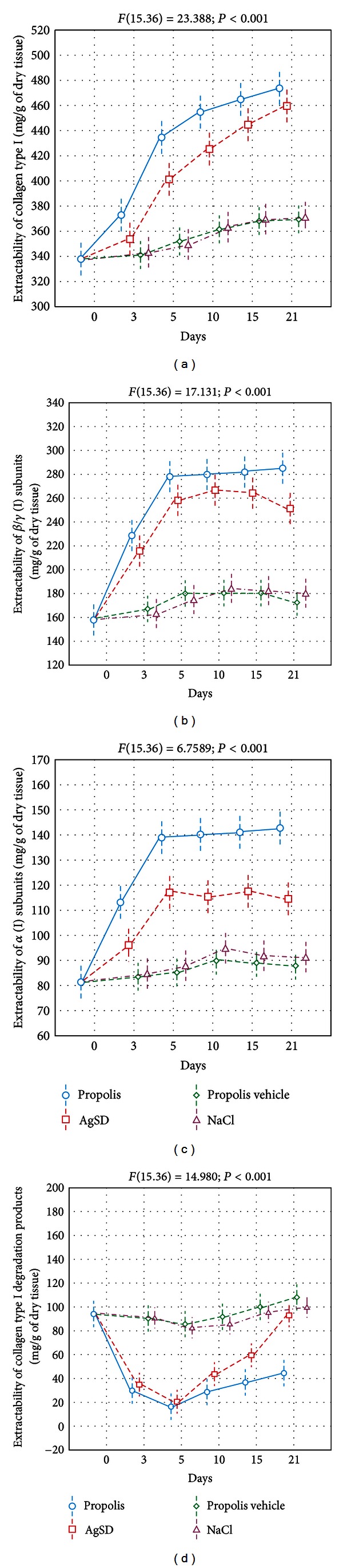
The extractability of collagen type I (a), *β*/*γ* (I) subunits (b), *α* (I) subunits (c), and collagen type (I) degradation products (d) from healing wounds (the 3rd, 5th, 10th, 15th, 21st days after burn infliction) treated with propolis, AgSD, propolis vehicle, and NaCl. Day 0—healthy skin. The data were estimated by ANOVA.

Tissue samples, taken from the healthy and wounded domestic pig skin on the 3rd, 5th, 10th, 15th, and 21st days after burn infliction, homogenized with acetone, suspended in acetic acid, and subsequently subjected to pepsin action were the source of collagen types I and III. The estimation of collagen types I and III extractability from the obtained material was performed by the application of surface plasmon resonance (SPR) method. According to observed collagen type I extractability, shown in Figure 1(a), the release of this protein from the injury matrix, reached the maximum level after propolis and AgSD implementation at the end of the study.

**Figure 2:**
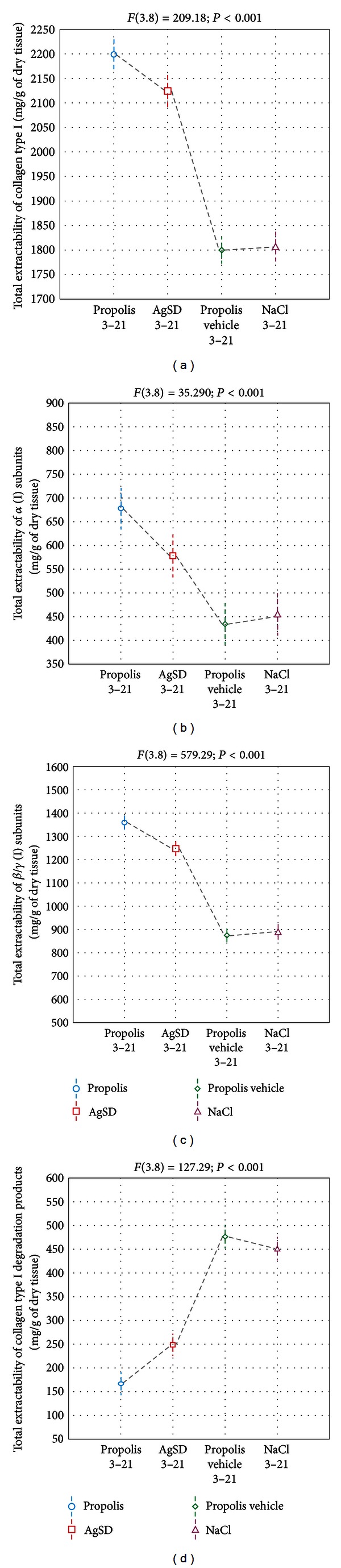
The total extractability of collagen type I (a), *α* (I) subunits (b), *β*/*γ* (I) subunits (c), and collagen type (I) degradation products (d) from healing wounds (since the 3rd up to 21st days after burn infliction) treated with propolis (3–21), AgSD (3–21), propolis vehicle (3–21), and NaCl (3–21). The data were estimated by ANOVA.

Thermal injury treatment with propolis resulted in higher release of components reacting with collagen type I antibodies, on the 5th and 10th days of the experiment, as compared with other applied agents (Figure 1(a)). In the case of NaCl and propolis vehicle treated injuries, the differences in collagen type I extractability between day 0 and the 21st day were not statistically significant. Furthermore, the apitherapeutic method of burns treatment, used in the present study, stimulated the highest, total extractability of collagen type I from burn wounds matrix (Figure 2(a)).

**Figure 3:**
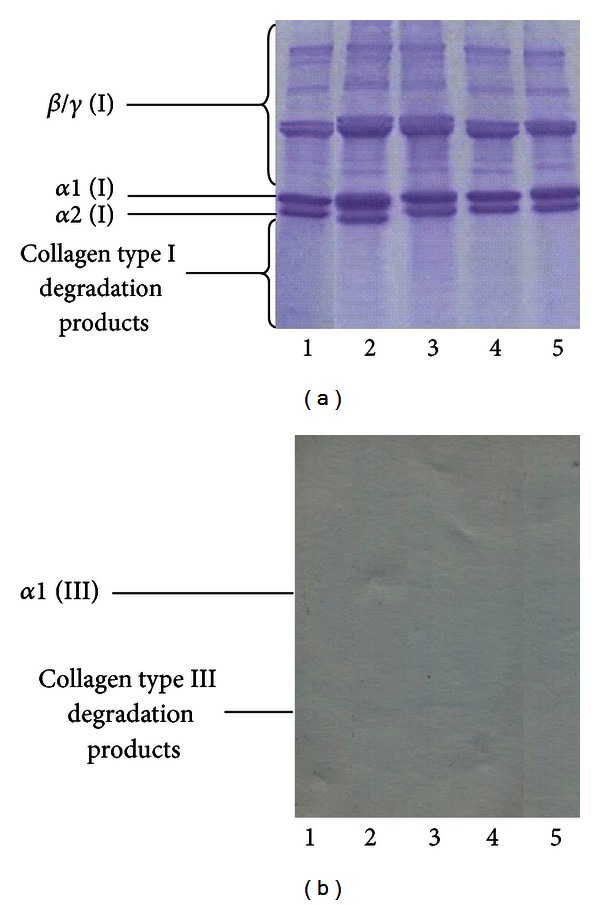
(a) Typical electropherogram of *α* (I) subunits, oligomers *β*/*γ* (I), and collagen type I degradation products, released from healthy and burnt skin. Collagenous components released from tissue samples using pepsin were submitted to 4–15% gradient SDS-PAGE, in nonreducing conditions. (b) Interferences of collagen type III, in electrophoretic profiles of collagen type I components extracted from healthy and burned skin. Collagen components were submitted to electrophoresis in the absence of dithiothreitol (reducing disulfide bonds) and subsequently—after electrotransfer to Immobilon—subjected to reaction with collagen type III antibodies. Lane 1: components of collagen type I isolated from healthy skin. Lane 2: components of collagen type I isolated from burned skin treated with propolis. Lane 3: components of collagen type I isolated from burned skin treated with AgSD. Lane 4: components of collagen type I isolated from burned skin treated with propolis vehicle. Lane 5: components of collagen type I isolated from burned skin treated with NaCl.

The electrophoretic evaluation of normal and burnt skin collagen type I revealed the presence of *α*1 (I) subunits, *α*2 (I) subunits, dimers of *α* chains (components *β*)/trimers of *α* chains (components *γ*), and heterogenic collagen type I degradation products (typical electropherogram is shown in Figure 3(a)).

Simultaneously, collagen type III negligible interferences (Figure 3(b)) presented above, obtained in nonreducing conditions, electrophoretic profiles of collagen type I components, were visible only in the case of the highest collagen type III extractability. The last observation was based on the immunoreactivity analysis of collagen III antibodies with components penetrating into the preparative gel, in the absence of disulfide bonds reducing agent.

Densitometric analysis of the received gels revealed that propolis and AgSD—during initial phase of the study—stimulated significant increase in *α* (I) chains and *β*/*γ* (I) subunits isolation (Figures 1(b) and 1(c)) with a subsequent stabilization of the mentioned components release. At the end of the study, burn management with the apitherapeutic agent resulted in maximal release of *α* (I) and *β*/*γ* (I) subunits. Moreover, as can be seen in Figures 2(b) and 2(c), propolis treated injuries exhibited the highest total extractability of *α* (I) and *β*/*γ* (I) subunits. The extractability of collagen I degradation products (Figure 1(d)), from propolis and AgSD treated wounds after preliminary decline, since the 10th day of the study presented a significant increase. On the other hand (Figure 1(d)), the maximum release of the mentioned products from wounds treated with NaCl and propolis vehicle was constant throughout the experiment. The latest methods of burn treatment resulted in the highest total extractability of collagen I degradation products, while the propolis stimulated the lowest release.

**Figure 4:**
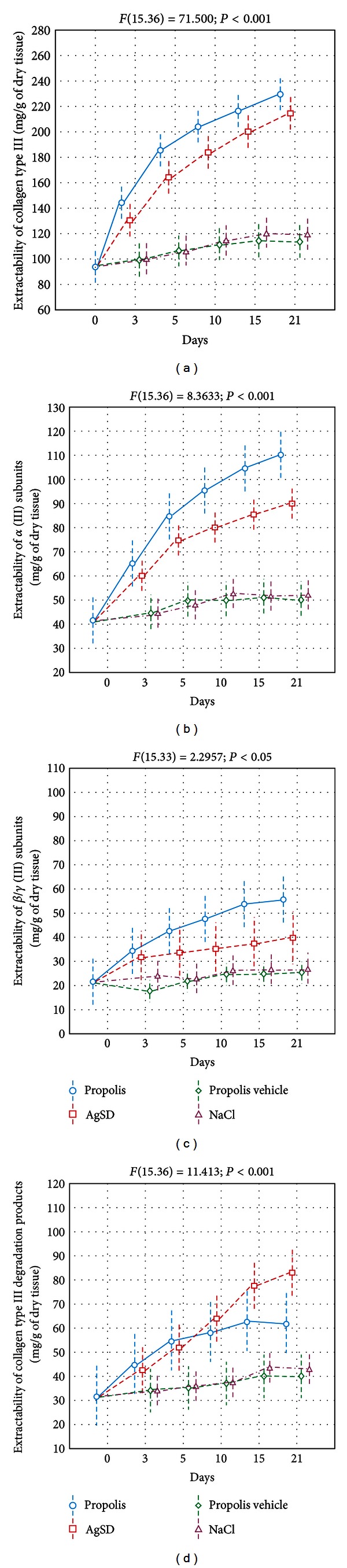
The extractability of collagen type III (a), *α* (III) subunits (b), *β*/*γ* (III) subunits (c), and collagen type (III) degradation products (d) from healing wounds (the 3rd, 5th, 10th, 15th, and 21st days after burn infliction) treated with propolis, AgSD, propolis vehicle, and NaCl. Day 0—healthy skin. The data were estimated by ANOVA.

As it was shown in Figure 4(a), the profiles of type III collagen extractability resembled those observed for collagen type I. However, propolis acted significantly stronger, than AgSD did, by stimulating the extractability of collagen type III and by contributing to the highest release of components reacting with collagen III antibodies on the 5th, 10th, and 15th days after burn infliction.

**Figure 5:**
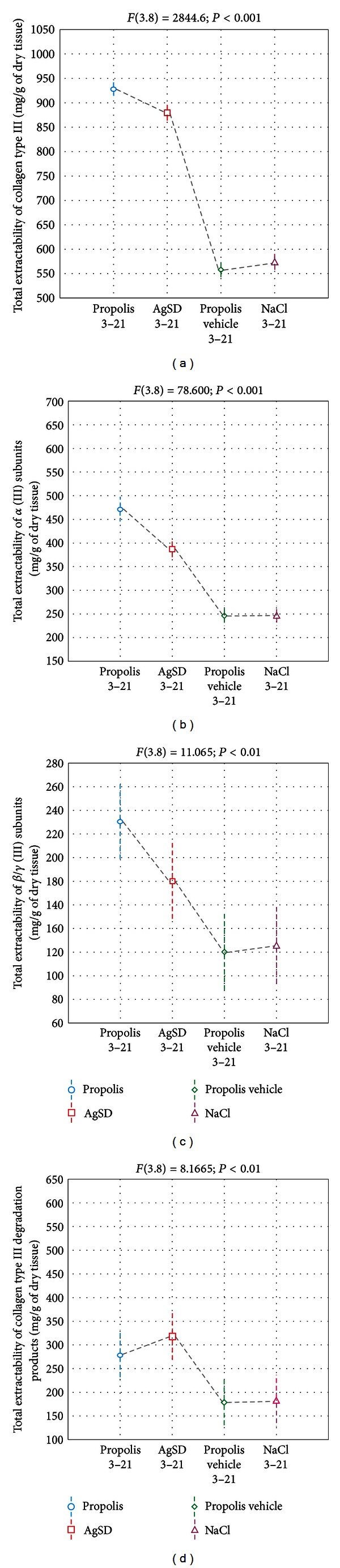
The total extractability of collagen type III (a), *α* (III) subunits (b), *β*/*γ* (III) subunits (c), and collagen type (III) degradation products (d) from healing wounds (since the 3rd up to 21st day after burn infliction) treated with propolis (3–21), AgSD (3–21), propolis vehicle (3–21), and NaCl (3–21). The data were estimated by ANOVA.

The collagen III extractability from NaCl and propolis vehicle treated injuries did not change throughout the whole experiment. Moreover, the apitherapeutic ointment stimulated higher, than other applied agents, total extractability of collagen type III from the burn wound beds (Figure 5(a)).

**Figure 6:**
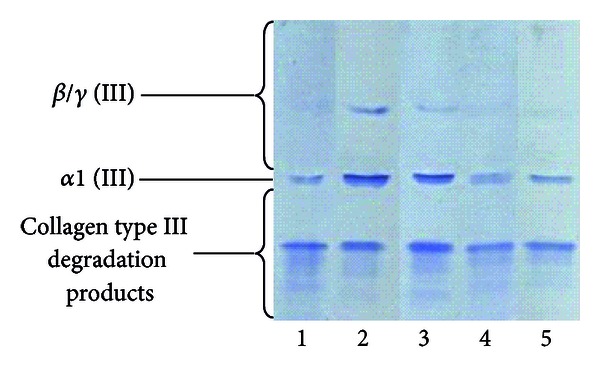
Typical electropherogram of *α* (III) subunits, oligomers *β*/*γ* (III), and collagen type III degradation products, released from healthy and burned skin. Collagenous components released from tissue samples using pepsin were submitted to 4–15% gradient SDS-PAGE, western blotted, and probed with collagen type III antibodies. Lane 1: components of collagen type I isolated from healthy skin. Lane 2: components of collagen type I isolated from burned skin treated with propolis. Lane 3: components of collagen type I isolated from burned skin treated with AgSD. Lane 4: components of collagen type I isolated from burned skin treated with propolis vehicle. Lane 5: components of collagen type I isolated from burned skin treated with NaCl.

The analysis of the obtained electrophoretic profiles revealed the presence of *α*1 (III) subunits, dimers of *α* chains (components *β*)/trimers of *α* chains (components *γ*), and heterogenic collagen type III degradation products (a typical electropherogram is shown in Figure 6).

Despite moderate qualitative differences visible in the obtained electrophoretic profiles, considerable quantitative variations characterize the extractability of collagen type III individual components from differently treated injuries. Regardless of the applied burn treatment method, the release of *α*1 (III) chains (Figure 4(b)) and, to a lesser extent, their oligomers (Figure 4(c)), directly involved in restoring of collagen fibers weave, corresponded with extractability of collagen type III (Figure 4(a)). Propolis wound treatment in comparison to other agents, AgSD, propolis vehicle, and NaCl, significantly enhanced both the extractability dynamics of *α* (III) chains and *β*/*γ* (III) subunits (Figures 4(b) and 4(c)) and the total release of the mentioned components (Figures 4(b) and 4(c)), which reflect the increased accumulation of collagen type III in the healing burns. On the other hand, attention should be paid to the intensified extractability dynamics (Figure 4(d)) and the total release (Figure 5(d)) of collagen type III degradation products from injuries cured with AgSD or propolis, as compared to wounds dressed with propolis vehicle and NaCl.

## 4. Discussion

Wound healing, physiological body response to injury, which is essential for the replacement of damaged structures with a subsequent restoration of skin integrity, can usually be divided into hemostasis, inflammation, proliferation, and maturation [11, 26]. Successful skin repair requires highly regulated activity of various cells which synthesize a wide array of cytokines, growth factors, and extracellular matrix molecules—including collagen [11, 27]. The last one, accumulated in the wound bed, influences the epithelial migration and proliferation at the beginning of the repair process, being also an integral molecule of properly tuned wound healing or pathological outcomes such as keloid and hypertrophic scars [7, 27, 28]. Therefore, to achieve a rapid skin rebuilding and scarring elimination, the implementation of new therapeutic agents modulating the healing process, including those of natural origin, is of a significant importance [7, 8, 29]. A popular natural remedy, propolis, is an excellent “candidate” for burn management [2, 7]. In contrary to the previous therapeutic factor, an agent of choice in the thermal injury treatment, silver sulfadiazine, is responsible for a number of troublesome side effects [9]. For a reliable assessment of the therapeutic activity of the mentioned agents on regenerative process, we applied for the first time a quantitative (using a surface plasmon resonance method) and qualitative (describing the profile of collagenous components) determination of collagen type I and III accumulation in the matrix of a thermal injury.

Significantly enhanced extractability, particularly at the beginning of the study, and a total release of collagen I from wounds treated with propolis confirmed the findings of de Moura et al. [30]. Moreover, we have found that the apitherapeutic agent more effectively, than AgSD, stimulates the expression of collagen type I—a key molecule in the course of tissue repair [18]. The increased collagen type I accumulation in the matrix of an injury cured with propolis may stimulate the repair process, since collagen I, an essential element of the skin connective tissue, is indispensable for the keratinocyte migration and reepithelization [18, 31]. Mentioned protein also interacts with matrix metalloproteinases (MMPs) influencing IGF-1 bioavailability, cell proliferation, modulation of inflammation, and bFGF releasing [26, 32, 33]. Furthermore, after initially intensified *α* (I) and *β*/*γ* (I) extractability from propolis treated wounds, the release of the mentioned components underwent noticeable stabilization, which reduces the risk of fibrosis [28]. A significantly higher total extractability of collagen type I *α* chains and their oligomers, from burns dressed with natural agent, which were found in the study, as compared to AgSD influence, corresponds with Iyyam Pillai et al's. results [11], describing the propolis potential in the tissue repair stimulation. The initial decrease in collagen I degradation product extractability from wounds treated with propolis and AgSD precedes the increase in releasing of the mentioned products, which may reflect the enhanced activity of MMPs and prevent uncontrolled excessive scarring and keloid development [34].

Found after propolis burn treatment, intensified collagen type III extractability may significantly accelerate the healing process, since the mentioned protein participates in hemostasis, by coagulation factors binding, influencing platelet aggregation and signaling activity or inducing thrombus formation [35–37]. Furthermore, collagen III forms a scaffold necessary for cell migration and regulates collagen I fibrillogenesis [38, 39], TGF-beta molecules signaling action, cellular contraction, differentiation, and the function of myofibroblasts, creating a regenerative niche for the progenitor cells [40]. The observed enhanced extractability of collagen type III and *α* (III) subunits particularly from propolis treated wounds, which correspond with previous studies [41, 42], may explain beneficial propolis reparative properties [1]. The increased accumulation of collagen III in fetal tissues in comparison to adult tissues led to the hypothesize that the mentioned ECM protein may favour the scarless phenotype of gestational wound healing [40]. Moreover, fetal wound healing is characterized by the absence or significantly reduced inflammatory phase with a subsequently accelerated reepithelization [43]. In fact, according to the suggestions of McLennan et al. [1], propolis is able to enhance the reepithelization process. Intensified extractability of collagen III and *α* (III) subunits (particularly at the initial stage of the experiment), being the highest in the case of propolis treated wounds, may result from its ability to stimulate the proliferation and survival of monocytes, cells indirectly enhancing the biosynthesis of glycosaminoglycans, elastin, and collagen type III [44, 45]. Our last findings may be the consequence of vitamin A [46] and vitamin C presence [47] in the composition of the applied natural therapeutic agent. Vitamin C is essential in collagen biosynthesis while vitamin A can control the preferential TGF-*β*3 expression promoting regeneration and scarless repair [45]. The unlimited accumulation of collagen III in the wound matrix may contribute to the development of keloids [48]. Therefore, it should be emphasized that the enhanced extractability together with total release of collagen III degradation products following AgSD and propolis implementation suggests the increased activity of MMPs which, in turn, determine a collagenous sequestration [49]. As we suggested in the previous paper [14], propolis influence on wound healing may resemble the action of another natural agent—glycyl-L-histidyl-L-lysine-Cu^2+^ (tripeptide-copper complex) described as a growth factor—enhancing the expression of ECM macromolecules, including collagens type I and III [6].

## 5. Conclusion

The presented results confirm the therapeutic efficacy of propolis which is connected with generating a favorable biochemical environment supporting wound healing process.

The implementation of the experiments aiming at exploring the differences between healthy and wounded skin may help to eliminate or reduce the development of fibrosis and hypertrophic scars significantly improving life quality of patients suffering from burns.
